# Improving patient notification of solid abdominal viscera incidental findings with a standardized protocol

**DOI:** 10.1186/s13032-014-0022-x

**Published:** 2015-02-04

**Authors:** Courtney E Collins, Nicole Cherng, Theodore McDade, Babak Movahedi, Timothy A Emhoff, Giles F Whalen, Jennifer LaFemina, Jon D Dorfman

**Affiliations:** University of Massachusetts Medical School and UMass Memorial, Worcester, MA USA

**Keywords:** Trauma, Incidental finding, Computed tomography, Abdominal CT, Quality improvement

## Abstract

**Background:**

The increasing use of computed tomography (CT) scans in the evaluation of trauma patients has led to increased detection of incidental radiologic findings. Incidental findings (IFs) of the abdominal viscera are among the most commonly discovered lesions and can carry a risk of malignancy. Despite this, patient notification regarding these findings is often inadequate.

**Methods:**

We identified patients who underwent abdominopelvic CTs as part of their trauma evaluation during a recent 1-year period (9/2011-8/2012). Patients with IFs of the kidneys, liver, adrenal glands, pancreas and/or ovaries had their charts reviewed for documentation of the lesion in their discharge paperwork or follow-up. A quality improvement project was initiated where patients with abdominal IFs were verbally informed of the finding, it was noted on their discharge summary and/or were referred to specialists for evaluation. Nine months after the implementation of the IF protocol, a second chart review was performed to determine if the rate of patient notification improved.

**Results:**

Of 1,117 trauma patients undergoing abdominopelvic CT scans during the 21 month study period, 239 patients (21.4%) had 292 incidental abdominal findings. Renal lesions were the most common (146 patients, 13% of all patients) followed by hepatic (95/8.4%) and adrenal (38/3.4%) lesions. Pancreatic (10/0.9%) and ovarian lesions (3/0.3%) were uncommon. Post-IF protocol implementation patient notification regarding IFs improved by over 80% (32.4% vs. 17.7% pre-protocol, p = 0.02).

**Conclusion:**

IFs of the solid abdominal organs are common in trauma patients undergoing abdominopelvic CT scan. Patient notification regarding these lesions is often inadequate. A systematic approach to the documentation and evaluation of incidental radiologic findings can significantly improve the rate of patient notification.

**Electronic supplementary material:**

The online version of this article (doi:10.1186/s13032-014-0022-x) contains supplementary material, which is available to authorized users.

## Background

The evaluation of trauma patients routinely involves the use of computed tomography (CT). With the frequent use of high resolution imaging comes increased detection of non-traumatic findings. Incidental findings (IFs) of the solid abdominal organs are among the most common discoveries. While they are usually asymptomatic and ultimately benign, they do present a risk of malignancy [1-4]. Solid abdominal organ IFs present a difficult dilemma for clinicians who must carefully balance the benefit of subsequent workup with the risks and costs of additional tests and procedures.

Management of these newly diagnosed solid organ lesions in trauma patients is complex. The high acuity and complexity of individual trauma patients who frequently present with multiple injuries and have multiple medical and surgical services involved in their care leads to prioritization of life-threatening and acute injuries first. Furthermore, the diagnosis and workup of IFs may simply not be feasible in the setting of an in-patient trauma service. Previous studies reported the prevalence of incidental CT scan findings. However the manner in which findings are approached by trauma teams has not been well-researched [5-9]. The little data that does exist have shown low rates of patient notification and low rates of follow-up regarding these findings even in the case of potentially serious discoveries [5,7,9-11]. This is especially concerning since trauma patients, like many Americans, may lack access to routine outpatient healthcare and therefore may not receive appropriate workup or education after hospital discharge. Finding a cost-effective way to approach these findings is important not only for trauma surgeons but for any provider who encounters IFs as to minimize dangerous clinical and medico-legal consequences.

We implemented a protocol dedicated to capturing and addressing IFs of the kidneys, liver, adrenal glands, pancreas, and ovaries in trauma patients at a Level 1 trauma center. We performed two retrospective chart reviews of abdominopelvic CT scans performed as part of a trauma evaluation to evaluate the frequency of IFs of the solid abdominal organs in this population and the adequacy of patient notification regarding these potentially malignant lesions before and after the implementation of an IF protocol. We hypothesized that a standardized approach would improve rates of patient notification for patients with abdominal IFs.

## Methods

### Study design and setting

This study was performed at a Level 1 trauma center in central Massachusetts. After approval from the University of Massachusetts Medical School institutional review board, a retrospective review of a prospectively-maintained trauma registry was performed to obtain a list of patients presenting as trauma activations between 9/1/2011 and 8/31/2012 (pre-protocol implementation) and 9/1/2012 and 6/1/2013 (post-protocol implementation). Trauma patients 18 years or older who underwent a CT scan of the abdomen and/or abdomen/pelvis as part of their trauma admission were eligible for inclusion. CT scans done both as part of an initial trauma evaluation and during the subsequent hospital course were considered. Patients who were discharged directly from the emergency department were excluded as the final CT read was not always available at the time of discharge. Patients admitted to services other than the trauma service were excluded as well. CT scans performed as part of the trauma protocol were typically, but not uniformly, without oral contrast. Use of oral and/or rectal contrast was reserved for cases in which hollow viscous injuries were to be ruled out. IV contrast was administered except in patients with elevated creatinine. All CT scans are read by an attending radiologist and reviewed by the trauma surgeon on call at our institution.

### Incidental finding protocol

Beginning in September of 2012, a clinical protocol was implemented for all IFs of the pancreas, liver, kidneys, ovaries, and adrenal glands. The patients were verbally informed of these IFs and the IFs were noted on their discharge summary. Each of these patients was then evaluated by a specialist during their trauma admission to determine if any further workup and follow-up was warranted. Necessary follow-up appointments were to be made prior to discharge. Prior to implementation of this protocol there was no system for approaching IFs, and each lesion was individually addressed at the discretion of the admitting attending and referred to the primary care physician for evaluation. Of note, the IF protocol applied only to patients admitted to the trauma service. Those evaluated by trauma but admitted by other services or discharged from the emergency department were not included.

Implementation of the incidentaloma protocol involved all members of the trauma team. The trauma surgical service includes residents, nurse practitioners and attending surgeons. The rotating residents receive an orientation prior to beginning their clinical rotation. As part of the orientation, they are introduced to concept of incidentalomas and the importance of notification and/or follow up. At least one of the 3 nurse practitioners are present daily. They assist with the tertiary surveys which include the review of finalized radiology dictations for incidental findings. The nurse practitioners were made aware of the pathway, given the contact information for the consultants and assisted with informing patients of incidental findings, ensuring that incidental findings were noted on the hospital discharge summaries along with the follow up plan. The trauma surgeons were also appraised of the protocol; one of the trauma surgeons (JD) would review the current trauma inpatient census for incidental lesions and notification of patients.

### Study protocol

Three trained reviewers abstracted all radiology reports for CT scans done during the two study periods. IFs were defined as any non-traumatic lesion of the liver, pancreas, kidneys, adrenal glands, and/or ovaries. Solid lesions, cystic lesions, as well as any finding that warranted radiologic or other follow-up as reported in the attending radiologist’s interpretation were included. Indeterminate findings on CT scans were reviewed by 2 attending surgeons (JD and JL) and then categorized as either likely traumatic or incidental. Traumatic lesions, normal anatomical variants, physiologic lesions, as well as any finding noted in previous radiology reports were excluded. Pulmonary, vascular, gastric, intestinal and bony lesions were also not included in this analysis.

For all patients with IFs, the electronic medical record (including inpatient and outpatient records) was reviewed for evidence of IF-related notification. Patients were considered notified if their discharge paperwork or dictated discharge summary contained information regarding the lesion or if there was evidence that the patient had seen the appropriate specialist during their trauma admission.

For all patients, demographic and clinical information including age, race, gender, injury severity score (ISS), and insurance status was extracted directly from the trauma registry.

### Data collection and statistical analysis

Study data were collected and managed using REDCap electronic data capture tools hosted at the University of Massachusetts Medical School. REDCap (Research Electronic Data Capture) is a secure, web-based application designed to support data capture for research studies [12]. Data was exported and analyzed using SAS 9.2.

Demographics and clinical characteristics of patients with and without abdominal IFs were compared using univariate tests of association. Specifically, t tests were used to compare continuous variables, while chi square tests were used for categorical variables except in the case of small cell sizes in which case Fisher’s exact tests were performed. Patient characteristics, IF frequency, IF anatomic distribution, and rate of patient notification were compared between the two study periods.

## Results

### Patient characteristics

A total of 1,117 patients underwent an abdominal or abdominopelvic CT scan as part of their trauma evaluation during the 21 month study period. 687 patients were seen prior to IF protocol implementation and 440 were seen after. Patients were predominantly male, white, and had an average age in the fourth decade of life. Patients with IFs were significantly older and more likely to have Medicare insurance compared to those without IFs (60.7 years old versus 40.5 years old). There were no statistically significant differences in race, gender or injury severity scores (ISS). Post-protocol implementation patients were older, had higher ISS, and were more likely to have no fault auto or Medicare insurance compared to the pre-protocol period. However, there was no significant difference in age, gender, ISS, insurance, or race between IF and non-IF patients in the pre- and post-protocol periods (Table 1).

**Table 1:** **Demographics for trauma patients undergoing abdominal CT scan Pre**- **and post**-**IF protocol implementation**

	**All patients**			**IF patients**		
	**Pre-** **protocol** **N = ** **687**	**Post-** **protocol** **N = ** **430**	**P value**	**Pre-** **protocol** **N = ** **140**	**Post-** **protocol** **N = ** **99**	**P value**
Age in years, mean (SD)	43.5 (19.7)	46.9 (20.8)	0.01	59.4 (18.8)	62.5 (19.9)	0.21
Male Gender, N (%)	489 (71.2%)	292 (67.9)	0.25	93 (66.4%)	68 (68.7)	0.71
Race, N (%)			0.4			0.99
White	561 (81.7)	335 (77.9)		121 (86.4)	84 (84.9)	
Hispanic	62 (9.0)	49 (11.4)		11 (7.9)	8 (8.1)	
African American	30 (4.4)	17 (4.0)		2 (1.4)	2 (2.0)	
Other	12 (1.8)	8 (1.86)		2 (1.4)	2 (2.0)	
Unknown	22 (3.2)	21 (4.9)		4 (2.9)	3 (3.0)	
Insurance, N (%)			<0.01			0.95
Private	105 (15.3)	59 (13.7)		24 (17.2)	8 (18.2)	
Medicaid	64 (9.3)	27 (6.3)		6 (4.3)	6 (6.1)	
Medicare	77 (11.2)	66 (15.4)		28 (20.0)	23 (23.2)	
No Fault Auto	346 (50.4)	248 (57.7)		66 (47)	43 (43.4)	
Self Pay	34 (5.0)	10 (2.3)		7 (5.0)	4 (4.0)	
Other	61 (8.9)	20 (4.7)		9 (6.4)	5 (5.1)	
Mean ISS (SD)	15.8 (11.6)	17.3 (12.0)	0.04	16.3 (11.4)	17.7 (11.8)	0.38

### Abdominal IFs

Of the 1,117 patients, 239 patients (21.4%) had 292 abdominal IFs. There was no significant difference in the frequency of IFs in the pre- and post-protocol period (140/20.4% pre-protocol vs. 99/23.0% post-protocol; p = 0.29). Overall, renal lesions were the most common IF (146 patients, 13% of all patients), followed by hepatic (95 patients, 8.4%), adrenal (38 patients, 3.4%), pancreatic (10 patients, 0.9%), and ovarian lesions (3 patients, 0.3%). There was no significant difference in the anatomic distribution of IFs across the two time periods (Table 2).

**Table 2:** **Anatomic location of incidental findings Pre**- **and post**-**IF protocol**

**Findings**	**Pre-** **IF protocol** **N** **(% all patients)**	**Post-** **IF protocol** **N** **(% all patients)**	**P value**
Any incidental finding	147 (20.4)	99 (23.0)	0.29
Renal lesions	87 (12.6)	59 (13.7)	0.6
Hepatic lesions	54 (7.9)	41 (9.5)	0.3
Adrenal lesions	25 (3.6)	13 (3.0)	0.6
Pancreatic lesions	6 (0.9)	4 (0.9)	0.9
Ovarian lesions	2 (0.3)	1 (0.2)	0.9

### Patient notification

A total of 31 patients with abdominal IFs (13%) died during their trauma hospitalization with no significant difference between the pre- and post-protocol periods (16 patients/11.4% vs. 15 patients/15.2%; p = 0.39). Rate of notification for surviving IF patients significantly increased by over 80% after the implementation of the IF protocol (p = 0.02; Table 3). When broken down by location, notification rate improved significantly only for patients with renal lesions. A trend towards improved patient notification was found in all anatomic locations, but this did not achieve statistical significance.

**Table 3:** **Percent surviving IF patients notified of finding by anatomic location Pre**- **and post**-**IF protocol**

**IF location**	**Pre-** **IF protocol**	**Post-** **IF protocol**	**P value**
	**(N = ** **124)**	**(N = ** **84)**	
	N (%)*	N (%)*	
All	22/124 (17.7)	27/84 (32.4)	0.02
Renal	6 /74 (8.1)	15/50 (30)	0.001
Hepatic	10/51 (19.7)	9/34 (26.5)	0.45
Adrenal	9/20 (45)	6/10 (60)	0.70
Pancreas	2/6 (33)	4/4 (100)	0.07
Ovarian	0/2 (0)	1/1 (100)	0.33

*% of patients with given.

## Discussion

Over 20% of patients with an abdominopelvic CT scan as part of a trauma evaluation had a new incidental lesion of their liver, kidneys, pancreas, adrenal glands and/or ovaries detected during our study period. Prior to the implementation of an IF protocol, less than 20% of patients with abdominal IFs were notified regarding their findings. A systematic approach improved rates of patient notification by over 80%. However, even though implementation of the protocol augmented notification, there is much room for continued improvement as still the majority of patients did not have adequate notification.

Our rate of IFs (21%) is lower than rates of IFs previously reported in the literature, which have ranged from 30-56% [6,7,9,11]. Our lower rate of IFs is likely because we restricted our definition to nonvascular lesions confined to the abdominal viscera whereas previous studies have applied a broader definition. In prior publications, many benign findings such as hiatal hernias were included. We decided to exclude these findings and focus on solid organ lesions that were malignant or potential malignancies as trauma patients frequently have multiple acute problems and providing a patient with a catalogue of new medical problems that are incidental and require no intervention would likely be overwhelming and unhelpful. Gastric and intestinal lesions were also not included as lesions of these organs may be incompletely defined on CT scan. Renal lesions were the most common finding in our cohort while pancreatic and ovarian lesions were relatively rare. Overall the rates of IFs in each anatomic location in our study were similar to those reported in other studies [6,7,10,11].

The average age of patients with abdominal IFs was more than 20 years older than patients without an IF. Other studies have also documented an association with incidental findings and advanced age [5,6]. The presence of an asymptomatic solid organ lesion in an elderly patient is especially complicated because clinicians must consider the practicality of initiating the workup for an asymptomatic lesion in a patient who may, for example, only have an additional 10 years of life expectancy or may not be medically fit to undergo the medical or surgical treatment for the lesion. Because the trauma population, like the population of the United States, is aging rapidly, IFs will likely become increasingly common in the trauma setting as their patient population gets older [13,14]. Establishing a way to integrate the care of traumatic injuries with other medical issues including IFs in these elderly patients will be an increasingly important issue going forward.

Due to the relatively small numbers and short follow-up, it is too early to determine whether any of the IFs in our study will turn out to be clinically significant. To date, no malignancies have been confirmed. Of note, there is ongoing debate regarding the proper management of asymptomatic IFs. Some lesions, such as renal cysts, have only a 1% malignancy rate and rarely require attention [15]. However, other findings such as pancreatic lesions carry a malignancy risk of up to 34% [1,3]. Because the risk of malignancy cannot be definitively excluded, the general recommendations for IFs of abdominal viscera is that they should be followed up with radiographic studies, laboratory testing, and possibly biopsy or operative excision for extremely suspicious findings [16-19]. Patient education regarding IFs and the need for follow up is important not only in terms of patient care but also to avoid potentially disastrous medico-legal consequences.

Prior to the implementation of our IF protocol, patient notification regarding incidental abdominal findings was poor. Less than 20% of patients with incidental findings had documentation of education related to their lesion during this time period. Importantly, patients with adrenal and pancreatic lesions which are the most likely to require post-discharge follow-up were notified about their finding less than half the time [3,17]. This is consistent with previously published literature which has shown low rates of patient education regarding incidental findings without a systematic approach. A study similar to ours found that only 27% of 211 of Pittsburgh trauma patients with incidental findings of the abdomen and pelvis had documentation of the finding in their paperwork [11]. Another study looking at 289 incidental findings in trauma patients found that only half the findings requiring attention prior to discharge were documented in the medical record [5].

Patient notification regarding incidental abdominal findings nearly doubled after the implementation of our IF protocol. Other studies have also shown that a systematic approach to IFs results in improved patient education [5,10,20]. The fact that many lesions captured in our study were deemed clinically insignificant or benign in the radiology dictation and did not require follow-up could explain why our post-protocol informed rate remained fairly low. In these cases lack of documentation could indicate that they were deemed not clinically relevant. This is supported by the fact that two categories with the lowest rate of patient notification after the implementation of our IF protocol were renal and hepatic lesions, which are most likely benign [15,21]. Additionally any verbal conversations with patients that were accompanied by documentation in the discharge paperwork would not have been captured by our chart abstraction.

Our protocol emphasizes notification of IFs while the patient is hospitalized. Other approaches to incidental CT findings have focused on notifying either the patient or the patient’s primary care physician after discharge, often in the form of a letter or other written communication. Inpatient notification was not always addressed, and in some cases actually declined [5,10,20,22]. We believe our approach, while logistically somewhat difficult, provides several benefits. First, we eliminate the need to locate a patient after discharge, which may be difficult if a patient does not live locally, is homeless, or was discharged to a rehabilitation facility, nursing home, or a family member’s residence. Additionally, addressing findings in the hospital allows for the creation of a plan for further workup, which may be more feasible for patients who are unable or unlikely to follow up after discharge for any reason. Previous systems have contacted the primary care physicians. However, many trauma patients do not have a primary care physician, and such a system requires that patients follow up with their primary care physician in a timely manner.

There are several important limitations for this study that need to be considered in the interpretation of the present findings. First, although all patients’ CT scan reads were reviewed, the scans themselves were usually not. IF data are dependent on the documentation of the attending radiologist. We assumed that major findings were consistently documented and that only minor findings were omitted from the report. Additionally it should be noted that not all patients received the same kind of CT scan. Slice thickness and the presence or absence of IV and PO contrast could impact the ability of a scan to detect a lesion.

Second, our definition of patient notification depends on documentation in discharge instructions or discharge summaries. Benign lesions may not be prioritized when completing discharge paperwork even if they were given attention by the trauma team. A system may need to be implemented for documentation of lesions that do not require follow up such as renal and hepatic cysts. Although many of these lesions may not require any further attention, they should still be explained to patients and documented in the chart. Any verbal communication from the team to the patient regarding findings on CT scans would not be captured without documentation on the discharge summary However if this is the case, lack of documentation of these conversations and findings is still concerning.

Finally, we were not able to determine the pathology of the lesions noted or the results of any subsequent workup so we are not able to comment on the benefits of IF documentation or the risks of poor follow-up. We also were not able to reliably report follow-up in the pre-protocol period as many trauma patients are from outside of our geographic area or seek care outside of our healthcare system and therefore do not have readily available outpatient records.

Although additional work remains to be done, we have shown that dedicated attention by the trauma team to abdominal IFs improves patient notification regarding these findings. In the multi-injured trauma patient, incidental lesions are easily overlooked or deemed to be insignificant when the short term survival of the patient is jeopardy. We recommend involving the entire team and designating one individual to champion this issue. Furthermore, periodic reminders and review of the process are required. Management of IFs is complex and represents a significant area for quality improvement. Additional attention needs to be paid to patients with IFs who are transferred to other services or who are discharged from the Emergency Department. Finally, all inpatient services should develop a strategy for approaching IFs in their own practice.

## Abbreviations

CT: Computed tomography
IF: Incidental finding
